# An Essay on Diabetes

**Published:** 1842-10

**Authors:** 


Art. XV.—*An Essay on Diabetes.* By H. Bell, d.m.p., one of the Librarians of the Faculty of Medicine of Paris. Translated by Alfred Markwick.—*London*, 1842. 8vo, pp. 96.

Although we have not had an opportunity of comparing Mr. Markwick's translation of Dr. Bell's little Essay on Diabetes with the original, we are so well pleased with the clear, concise, and compendious sketch which Mr. Markwick has supplied, as to be disposed to believe that he has falsified the assertion that "Every one suffers by a *translation* excepting a *bishop*." He has, in fact, given us a taste of, or a reference to all the honeyed water that has been known to flow for the last 2000 years and upwards, beginning with Hippocrates, b. c. 400, and hunting down to Mr. Smith, a. d. 1841; for in a small work of only ninety-six pages, teeming throughout with citations and references, five of them consist of a closely-printed enumeration of "those works which have appeared to him useful to consult, and capable of throwing light on some parts of the history of diabetes." Mr. Markwick has, in short, furnished us with a hive of diabetic honey, collected from a multiplicity of select literary flowers. *Sic vos non vobis mellificatis!*

Notwithstanding the researches of the most able physicians of this and of other countries, it is much to be regretted that no satisfactory information on the nature and mystery of the disease in question has hitherto been communicated. We have looked through all that Mr. Markwick has laid before us, in the anxious hope of discovering some mitigation of this opprobrium, but in vain. The theories which have appeared the most plausible have been briefly stated; the chemical analysis of the urine in the different species of diabetes has been given; the symptoms which characterize the disease have been defined; and the most successful modes of treatment enumerated; but, as our author observes:

> "All the hypotheses made on the nature of diabetes are still unsatisfactory; because the subtlety of mind is not sufficient by itself to discover the truth, and because we are still too ignorant of several points in the history of this disease to be able to find out its proximate cause." (p. 52.)

We closed the pithy little essay before us, therefore, with only the satisfaction of having refreshed our memory by a perusal of all that has hitherto been advanced as to the etiology, pathology, and therapeutics of diabetes, but without acquiring any additional information as to the mode of curing it. As, however, our experience in the methodus medendi entirely coincides with that of Dr. Bell, we shall quote his translator's own words:

"We have seen that the animal regimen, opium, warm-baths, and blood-letting were the most efficacious remedies in relieving the severest symptoms of diabetes. It is always useful to combine them together, and not to trust to one only. Thus, opium, animal diet, and warm-baths have always succeeded, in Dr. Bardsley's hands, if not in curing, at least, in greatly alleviating the symptoms. Others add tonics and astringents, when the stomach can bear them. It is only in those cases where these means prove useless that we are authorized in having recourse to others of certainly much less efficacy." (p. 60.)

